# The Problem of Effort Distribution in Heavy Glycolytic Trials with Special Reference to the 400 m Dash in Track and Field

**DOI:** 10.3390/biology11020216

**Published:** 2022-01-29

**Authors:** Antonio Cicchella

**Affiliations:** 1 International College of Football, Shanghai Tongji University, Shanghai 200092, China; antonio.cicchella@unibo.it; Tel.: +39-339-3355886; 2 Department for Quality of Life Studies, University of Bologna, 47921 Rimini, Italy

**Keywords:** effort, exhaustive exercise, glycolytic metabolism, termination of effort, fatigue endpoint, teleanticipation, pacing strategies

## Abstract

**Simple Summary:**

Short, intensive, but above lactate threshold physical trials or competitions which last <1 min but are close to this time limit, are on the border between glycolytic and aerobic efforts. The distribution of effort is critical in these tasks to achieve the best possible results. However, it is still unclear which of the general rules, descending from the theory, should be adopted by athletes (or any other subject facing a similar task). The 400 m dash competition of track and field has been taken in this systematic review as a paradigm for surveying the determinant factors which influence the pacing strategy and the end of effort. From the literature it emerges that there are several approaches, grounded in the philosophical basis of effort, which determine effort distribution. The problem is still open and a clear direction has not yet emerged from the available studies on the topic.

**Abstract:**

Background. Biological factors are ultimately responsible for the cessation of effort in short, maximal glycolytic efforts. However, how these factors are sensed by the brain and act in a loop or feedforward way to regulate the distribution of effort is still unclear. Methods: A systematic review of existing literature on short term glycolytic exercise has been performed on publicly available databases (Google Scholar and Pudmed). Results: The problem of effort termination in fast maximal glycolytic activities after 100 years of research is still open. It is not clear if a central governor of effort exists, if the limitations are in the energy transport and utilization system, or in the psycho-social factors. Conclusions. The solution probably resides in a mixture of factors, but how the different components interact is still a mystery for science due to the philosophical grounding of the experimental approaches.

## 1. Introduction

Glycolytic anaerobic metabolism was indicated as the limiting factor in several sport activities, and it was experimentally linked with fatigue and performance cessation. The limiting factors in humans, according to Nobel prize holder Arthur V. Hill [1], reside in the O_2_ transport system [2]. The role of volitional characteristics in determining the limits of performance has been evidenced, but it is not still clear [3,4] how it works in athletes. Because of the relatively recent interest of scientists in volition in sport, there is not a well-established base of knowledge on the topic [3]. The three main theories of volition are the theory of action control (ability to apply self-regulation strategies), the Rubicon model of action phases (the achievement of a point of no return which obligates one to continue the performance), and the strength model of self-control (volition) [3]. However, these three theories come from the general psychology domain and have been adapted to sport. Sports, by definition, are a broad range of activities, which are very different from each other. In sport activities, the process of fatigue in the performance of comparable duration and intensity (cycles per minutes) are very different among disciplines as they are affected by several factors. While in a long-distance run, a model such as the Rubicon model of action phases can work because there is sufficient time to think and adopt, adapt and/or change the running strategy, in short events the time for thinking is limited. In sport, a different specific explanation for the sustainability of physical effort and/or effort termination has been advocated. One performance where glycolytic pathway is mostly stimulated with less influence of any other confounding factors is during the 400 m dash run in track and field, thus, this can be assumed as a paradigm for maximal anaerobic effort [5]. Of special interest from a practical point of view, is the pacing strategies employed in the run. Until now, different recommendations for practice have been developed by scientists, grounded in different theories of fatigues.

However, many obscure points remain about the reasons for termination of effort, and about the existence of a central governor in the brain for effort termination. In addition, the mechanisms for the integration of peripheral signals of fatigue and their raising to conscience awareness are not clear and, to some extent, have not yet been explored. Thus, it is useful for practical purposes of training to explore the existing knowledge on effort termination and planned behaviors in heavy glycolytic efforts.

The aim of this systematic review is to consider the existing theories on limiting factors in exhaustive performance, using the optimal pacing strategies in the 400 m dash of track and field as a paradigm.

## 2. Materials and Methods

**Figure 1:**
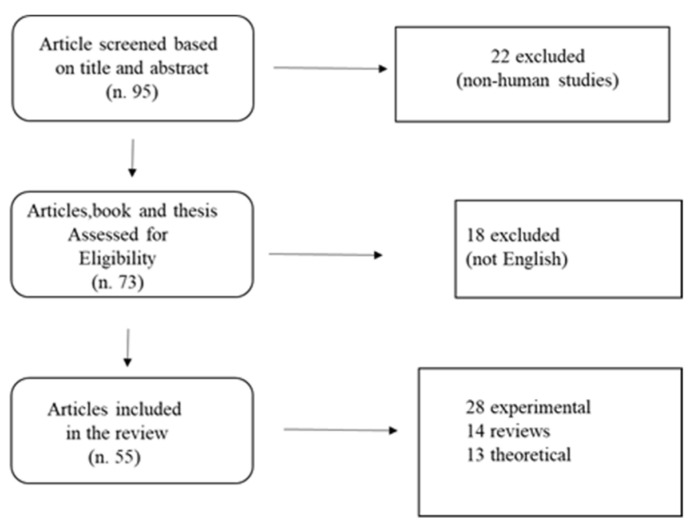
Flow chart of the search strategy.

A search of existing literature was undertaken using the databases Pubmed and Google Scholar in order to find research on the topic which met the following criteria: written in English (only two fundamental papers in German has been included), be experimental reviews or theoretical, dealing with sub-elite or elite athletes. The exclusion criteria were: papers dealing with animal models and written about non-high-level athletes. A search in other databases (Psychinfo, SportDiscus, and Web of Science) gave the same results. The following search terms were employed in the time range 21 December 2021 and before: 400 m race, fatigue, run endpoint, maximal effort duration, end of performance, anaerobic fatigue, maximal effort, glycolytic fatigue, termination of effort, decline of performance, physical exhaustion, knowledge of results, teleanticipation. Additional search phrases were: “termination of effort with heavy lactate accumulation”, “heavy glycolytic trials”, and “muscle contraction impairment”. After the removal of duplicates, a total of 98 papers were identified. The titles and abstracts of the papers were read, and on the basis of the selection criteria, relevant articles were retrieved for review. In addition, the reference lists from both the original and review articles retrieved were also reviewed. A total of 55 papers met the inclusion criteria and were included in this systematic review. This review was performed in accordance with the PRISMA (Preferred Reporting Items for Systematic Reviews and Meta-Analyses) guidelines. The search strategy is reported in Figure 1.

## 3. Results

### 3.1. Acidosis (pH), Lactic Acid (LA) and Limitations to Anaerobic Glycolytic Performance

The main explanation for the cessation of effort, which is historically given in the absence of other evidence, is that the acidosis likely deactivates the enzymes needed for energy production in the muscle. Peripheral blood lactate levels of 20.5 and 18.9 (±0.5) mm/L have been recorded 7 min post-race in Caucasian and African 400 m dash runners, respectively [5]. In another study, the measurement of muscle pH in the gastrocnemius of four subjects following a 400 m timed run on the track averaged 6.63 ± 0.03, while blood pH and HLa were 7.10 ± 0.03 and 12.3 mM [6]. The consumption of H+ ions resulting from the split of ATP or the depletion of phosphocreatine in working muscles [7] is the cause of acidosis. Lactic acid is toxic for the nervous system, causing dizziness, vision’s disturbances, and vagal reflexes (vomiting) at high concentrations [8]. The idea that limitations to performance happen mostly inside the muscle was supported by studies investigating the peripheral nervous system with Emg methods. Such studies showed that the causes of the end of performance are prevalently biochemical and happen in the working muscle [9]. Considering the 400 m run trial split into two 200 m segments, when observing the behavior of finalists (2017) of world IAAF (International Association of Athletics Federations) championships, both males and females showed an increase of speed up to 200 m, after which there is a remarkable linear decline of speed which continues until the end of the race [10]. The slope of this decline is more marked for males than for females. Equations considering the bioenergetics of the 400 m run have been proposed to model this drop in performance [11] and it has been found that the critical distance for running at maximal speed is 291 m. According to the mathematical model of Keller, until 291 m the race can be run at maximum propulsive speed, after this threshold, a distribution of effort must be adopted [11].

The equation proposed by Keller, from a model based on Newton’s second law is as follows:

dv dt + vτ = f(t)(1)

where v is the runner’s speed as a function of time t, τ is a constant characterizing the resistance to running (air, friction, proportional to running speed), and f(t) ≤ F is the propulsive force per unit mass. Empirical knowledge of human exercise physiology is expressed in the assumed relation between propulsive force and energy supply,

dE dt = σ − fv,(2)

where E represents the runner’s energy supply, with a finite initial value E0, and is replenished at constant rate σ. In spite of this replenishment, the energy supply reaches zero at the end of the race. τ, σ, E0, and F were found by comparing the optimal race times to the existing world records for 22 race distances ranging from 50 yd to 10,000 m [12]. A later study of energetic equations by Reardon [12] introduced an “X-factor” into the model, this refers to the rate of removal or disappearance of metabolites (e.g., Lactic Acid) and gives a more accurate prediction of split times in the 400 m. Reardon concluded that the best pacing strategy must include two phases: an acceleration phase, limited by the runner’s inertia and without any influence of catabolites, with monotonically increasing velocity, followed by a deceleration phase, limited by X-factor accumulation, with monotonically decreasing velocity [12].

### 3.2. Pacing

**Figure 2:**
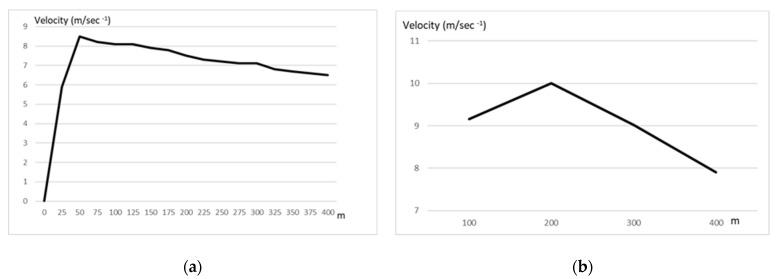
Velocity profile in the 400 m dash, plotted every 25 m or every 100 m. (**a**) data from elite athletes every 25 m [19]. (**b**) data from elite athletes every 100 m [20].

The idea of pacing in the fastest runs of track events is not new, and its popularity among coaches must be ascribed to the seminal work of Carlo Vittori in the 1970s and 1980s [13,14]. Models based on individual physiological (e.g., oxygen consumption, maximal speed, and performance in shorter distances e.g., 300 m) and psychological (albeit these characteristics have not been specified) characteristics were proposed for an optimal strategy [13] considering some individual differences. However, other possible parameters (e.g., enzymatic profile of the athletes) have not been investigated. Three different pacing behaviors can be observed: a positive pacing, which means that the second half of the competition is run with a higher time, negative pacing, which means that the time in the second split is decreasing, and an even pacing strategy. Given that the practical (for the field) determinant factor for a successful 400 m trial is the optimal distribution of effort, which also influences tactical choices [8,9], there were many studies which investigated the optimal distribution of effort in the 400 m race [10,12,13,14,15,16,17,18]. If we consider splitting the 400 m into two 200 m segments, or in shorter splits as depicted in Figure 2 (25 m), we can observe that after the first 50 m the velocity starts to decline. In addition, a different behavior can be noted in elite and sub-elite athletes.

As shown in Figure 2, the decline in velocity happens just after the first 50 m, if we consider the speed every 25 m as reported by Pollit [19] or after 200 m if we consider the 100 m segment [20].

With respect to oxygen consumption, a relatively high V˙O2 can be reached within 25 s from the start of a 400 m trial and the end V˙O2 values may be the consequence of metabolic perturbations correlated to the acidosis [21]. The athlete has to arrange their energy consumption per unit time with respect to the finishing point.

The first 200 m of the 400 m race was run by world-record-setting athletes with a speed ranging from 90 to 95% of their personal best in the 200 m race [15]. Mathematical [11,12] and even economical-mathematical [17] methods of investigation have been explored to model the effort distribution in the 400 m dash race. Mathematical models taken from the microeconomics, assuming that the limiting factors happen in the second half of the race, concluded that the choice between positive, negative or even splitting depends on the physiological characteristics of the athlete [17]. Thus, unlike the physics approach which gives general formulas [11], this approach came to the same conclusions as field-based studies [13]; there are individualized strategies based on physiological characteristics. Data on time splits of high-level athletes are difficult to harvest, due to the paucity of the population itself, and of measurement issues (location, high level competitions). Some publicly available biomechanical reports provided data about time splits in the 400 m race [19,20] (Figure 2). All athletes, both males and females, utilized a positive strategy. Usually, studies on pacing in the 400 m consider the split times in the first and second 200 m of the race [14,17], for practical reasons linked to the practical feasibility of the measurements on the field, while the more detailed analysis considered split times every 25 m [19]. A study assessing the relationship between the first 200 m pacing strategies and the final result in the 400 m [13], showed that the optimal pacing for the first 200 m, was equal to 93% of the time of athlete’s personal record in the 200 m, compared to 95% and 98%. The ratio of 93% to maximal 200 m performance also showed the speed at which the differential between the two halves of the race was smaller. However, this result was not obtained in a competition setting, which has been shown to strongly influence the pacing strategies. A seminal study using a mathematical model of the energy production/dissipation in the 400 m to determine the optimal pace strategy concluded that the optimal pacing strategy for the 400 m is a positive one, e.g., to run the first 200 m much faster than the second 200 [22], and not to try and adopt an even strategy, as indeed is suggested in longer distances [10].

When considering an effort of similar duration, the 1000 m race in speed skating, which lasts about 1 min and 6 s, it was demonstrated should be performed in an all-out fashion and not with a uniform velocity after the start [22]. However, in speed skating the motion is totally different from running, as the acceleration not so abrupt as in running due to the need of overcome friction.

Another interesting contribution came from the micro-economic theory, which suggested a different approach, e.g., individualized and based on best performance and previous races times [17]. Even in this case, for the 400 m dash a positive split is the recommended strategy. When 100 m intervals were considered (analyzing the trials of 400 m in 154 runners), it was found that the critical point is to maintain the speed in the second and third 100 m segments [23].

### 3.3. Teleanticipation

Pacing strategy has been proposed to be a teleanticipatory system, that is, a marker of underlying physiological systems, in which the brain anticipates the end point of exercise, and thus regulates the pace to avoid catastrophic derangements to homeostasis [24,25,26]. Anticipation concerns not only the basis of harmonic, biomechanically optimized motion, but also a teleanticipation for the optimal arrangement of exertion, which avoids early exhaustion before reaching a finish point [26]. The seminal work of Ulmer [25,27] modelled the teleanticipatory system as loop between afferent and efferent signals to/from the muscle which is mediated by the SNC in order to regulate the biomechanical and biochemical parameters of motion to optimize performance. Among basic electrochemical parameters, ETC (electron transport capacity) and redox potential have been shown to have a significant role [25]. This hypothesis was demonstrated by experiments in which the subjects did not know the end point of exercise and thus adopted different anticipatory strategies to avoid exhaustion and harm to homeostasis while optimizing performance [23]. The teleanticipation was postulated to have physical correlates in a central governor [28] (the so-called central governor model-CGM), a brain structure or a network of brain structures which integrates the afferent signals and regulates the strategy of pacing in an unconscious way, with the aim of maintaining homeostasis during running, according to the original idea of Ulmer [27] which was later developed by other authors [29,30]. However, the role of a central governor of exercise in the brain, has been questioned [31]. Shepard [31] states there is no evidence of such a center in the brain, and that self-regulation of pacing strategies happens as automatic responses to the exercise demands. Shepard argues that the CGM model is in contradiction with the Hill model of a “brainless” cardiovascular model, which grounded the exercise metabolism in mechanical regulations out of the brain’s integration control. The existence of a central governor of performance, has also been questioned by other authors [32] who proposed an alternative model based on motivation. This idea is not new. In the 1970s, the renowned physiologist Per Olof Astrand [33], proposed that, beyond any physiological limits, the will was the limitation for anaerobic lactacid performance, thus demanding the effective limits of performance to volitional factors. However, motivation is an entity which is difficult to quantify. An elegant synthesis was proposed by Edwards [34] which states that brain regulation of exercise operates at different levels of consciousness, such that minor homeostatic challenges are addressed automatically without conscious awareness, while larger metabolic disturbances attract conscious awareness and evoke a behavioral response. On the contrary to unconscious, motivational, and purely physical, an ecological behavioral approach was proposed assuming that decision-making (e.g., the pacing strategy) is a behavioral expression of the perception of affordances. This theory proposes that during natural interactive behavior at the neuropsychological level, action-selection and specification are one and the same dynamic process [35]. In support of this hypotheses some experimental work showed that the knowledge of the duration of effort influences the rate of perceived exertion (RPE). Athletes who run for 10 min, that were suddenly informed that their trial would be continued for other 10 min, scored higher in RPE between the 10th and the 11th minute of continuation in comparison to subjects who knew their trial would be 20 min long. They also showed a change in the focus of attention from external to internal, and showed a worse affective score which was more negative [36]. However, this study was performed using an aerobic protocol, and there are no similar studies employing anaerobic protocols. The concept of teleanticipation in anaerobic conditions, is determined by objective markers of anaerobic fatigue and thus is strictly linked to the concept of anaerobic threshold (AT). Further, anaerobic threshold is a concept which has been recently revised in a seminal paper by Poole et al. [37], which poses several issues on the origin and significance of AT, especially on the role of lactate in AT sensing. Lactate is, in fact, a major responsible of perceived exhaustion and the relationship between lactate and exhaustion is indifferent from training status and gender [38]. Besides lactate, during exhaustive exercise (aerobic) glycogen is depleted in the cortex, hippocampus, and brainstem probably due to a specific neuronal activity [39] mainly located in astrocytes cells [40].

### 3.4. Brain and Perception of Effort

From experimental studies on runners, it seems that the brain uses a scalar timing mechanism to predict the end of a performance [41,42]. A standard measure of perceived exertion (the Borg RPE visuo-analog test [43]) scales with the proportion of exercise time that remains [41]. In fact, it has been shown that comparing the same runners in a half and full race of 13.1 miles, the RPE expressed against time did not change [41]. The same effect was observed in cycling [42]. In shorter efforts, lasting 15 to 240 s, has been shown that the major sensation of effort comes from the working muscles while central sensations from the body made only a relatively small contribution to overall effort perception [44]. This finding has been confirmed in another study which employed a 100 s isokinetic leg extension task, where the subjects informed of the endpoint time showed less fatigue than the ones who did not know the end point [45]. A similar experiment using a Wingate test showed a reduction in power output when the subject was deceived on the duration of the trial. Subjects who believed the trial was 30 s long (instead of the real 36 s) reduced their power output in the last 6 s, while those who did not believe this did not reduce their power output in the last 6 s of the 36 s trial. This was interpreted as the presence of a preprogrammed 30-s “endpoint” based on the anticipated exercise duration from previous experience [46]. Knowledge of the endpoint dealt to a conservative pacing strategy in longer distances (30 km) cycling, with a major activation of the prefrontal cortex in the brain, e.g., more thinking [36]. However, the fatigue resulting from a 30 km race is not qualitatively comparable to anaerobic glycolytic fatigue.

However, deception studies have been questioned, because the variables that can be manipulated (distance, time, cadence, environment, feedback, verbal encouragement) are manifold and there are few homogeneities in the studies which used deception techniques to study athlete responses [47]. The drops in power observed in the 30 s deception experiments solely reflect the drop in cadence, e.g., rhythmic capacity which is regulated by the brain. The rhythmogenesis of movement seems to be a subjective characteristic, which can be investigated and identified by using specific sensory-test batteries for rhythmic capacities [48]. However, behavioral anticipatory systems are widely diffused in nature. According to the definition of Robert Rosen, “An anticipatory system is a natural system that contains an internal predictive model of itself and of its environment, which allows it to change state at an instant in accord with the model’s predictions pertaining to a later instant” [49,50]. The model of Rosen [49,50], is based on “information”. As demonstrated experimentally [36,47,51], the athlete seems capable of constructing an internal surrogate for time as part of a model that can indeed be manipulated to produce anticipation, and this internal time runs faster than real time. The runner has a model of the future but does not have a definitive knowledge of the future itself. The present change of state is determined by an anticipated future state, working as a feedforward system [49] according to a model which is believed to be optimal for the task to be accomplished which is ultimately an encoding and decoding of information. This information comes from the internal (thinking, memory, efferent and afferents signals, diminished carbohydrate availability, elevated serotonin, hypoxia, acidosis, hyperkalemia, hyperthermia, dehydration and reactive oxygen species, lactate levels, maximal cardiac output [51]), and from the external (temperature, wind, surface, crowd, other competitors). Faulty encoding leads to faulty models of performance [49]. In addition, it has been noted that previous subjective conditions strongly influence anticipatory thinking [51] as well as differences in racing against an opponent compared to time-trial exercises. Relational aspects have been postulated to outweigh the benefits of the individual optimal energy distribution [52,53,54]. Racing against an opponent has proven to benefit pacing only in some conditions. When an opponent is present, the focus of attention changes from internal to external, thus improving the management of fatigue [55,56]. In a race, the opponent(s) can be considered as a social invitation to performance and thus pacing [52].

## 4. Discussion

Anaerobic glycolytic “fatigue” is different from fatigue experienced in longer efforts. It is not a gradual accumulation over a long period of time. Instead, it is a rapid and sudden drop in running capacity, as experienced by the runners of the 300 and 400 m dash, when a certain time (<1 min) and distance, 291 m) is achieved. From this point onwards, the legs’ muscles are unable to work at the same amplitude and pacing as before. In addition, time for cognitive processes is limited due to the shortening of the effort. Unfortunately, available technology cannot measure the muscular (EMG), metabolic (lactate can be measured only at the end of the trial), and nervous (brain EEG can be measured only in laboratory conditions and not in running) activity during the effort with a reasonable precision, and these factors cannot be replicated with an acceptable standard of similarity to track conditions on a treadmill. It is also not possible to measure RPE during a 400 m without altering running mechanics. Thus, all the inferences are speculative as they are extrapolated from the split times and biomechanics of running. The lactate by-product of glycolysis has been reconsidered as a source of energy and is toxic for several organs (skeletal and cardiac muscles), impairing contraction, the kidney function, and the nervous system. Strategies to avoid lactate intoxication while maximizing the performance, are the basis of the pacing strategy in the 400 m dash run. The pacing strategies (<1 min, or for some authors shorter) in high intensity, exhaustive running are proposed to be dictated by a teleanticipatory system, which integrates peripheral (muscle fatigue) and central (reasoning, memory, rhythmic capacities) processes. This system acts at different levels, from basic reflexes to conscious processes. At the present state of the art, it is unclear from the literature if a central governor which regulates the process of pacing exist. It is also unclear if some process happens first locally and then centrally, if the limit is in the cardiorespiratory system and metabolic acidosis (systemic and in the muscle) external (social) environment, motivation or it is a learned behavior of decision making based on previous experiences. Results from the research are controversial and further investigations are needed.

## 5. Conclusions

There is no convincing evidence to support or to contradict the existence of a CGM which regulates anaerobic glycolytic pacing effort. Thus, a behavioral perspective seems the most suitable to explain the pacing strategy. As a practical recommendation, it emerges from the study of existing relevant literature, that a tactical approach based on the contingent characteristic of the race (environmental conditions, previous eliminatory trials, knowledge of competitor´s strategies) and an individual athlete´s characteristics (endurance or sprint-oriented athlete) are the major determinants of success in the 400 m dash in track and field. However, the paradigm of mechanisms of exhaustion in heavy glycolytic trials can be extended to other human activities. These considerations can be extended, with a certain degree of accordance, to other activities requiring exhaustive glycolytic maximal effort, for example, in heavy physical work or in military operations. Further studies are necessary to elucidate the underlying mechanism of effort cessation because the literature is still unclear about the fundamental basis of fatigue. Furthermore, the existence of a central governor of effort based in the higher brain structures is still debated and unclear.

## Data Availability

The study does not report any data.
